# Sialidase Neu3 Induced Sialic Acid Disorder and Promoted Vascular Endothelial Injury Through Transcription Factor SP3

**DOI:** 10.1111/jcmm.71169

**Published:** 2026-05-12

**Authors:** Yining Lu, Peng Xiang, Qingqiu Chen, Xianmin Wang, Le Chen, Tongchuan Wang, Rong Hu, Limei Ma, Chao Yu

**Affiliations:** ^1^ College of Pharmacy Chongqing Medical University Chongqing China; ^2^ Chongqing Key Laboratory for Pharmaceutical Metabolism Research Chongqing China

**Keywords:** atherosclerosis, endothelial injury, NEU3, Neu5Ac metabolism, transcription factor SP3

## Abstract

Atherosclerotic cardiovascular disease is still a major cause for the increasing mortality worldwide. The elevated metabolite N‐acetylneuraminic acid (Neu5Ac) was considered one of the important risk factors responsible for the initiation of endothelial impairment, then participated in atherosclerosis progression. However, the reason for inducing Neu5Ac accumulation as well as its underlying mechanism remains unclear. Here, we applied human umbilical vein endothelial cells (HUVECs) and ApoE^−/−^ mice to investigate the key genes responsible for Neu5Ac accumulation as well as its regulation mechanism. We found that tumour necrosis factor alpha (TNF‐α) could induce Neu5Ac metabolism disorder and increase Neu5Ac level, accompanied by the increasing levels of inflammatory markers in HUVECs. Further, we found that sialidase NEU3 was significantly up‐regulated, accompanied by Neu5Ac accumulation. NEU3^siRNA^ in HUVECs could significantly reduce Neu5Ac levels and alleviate endothelial inflammation. In contrast, the addition of exogenous Neu5Ac reversed such phenomenon. Furthermore, we noted that NEU3 was highly expressed in atherosclerotic plaques from ApoE^−/−^ mice as well as atherosclerotic patient tissues. Mechanically, we found that the transcription factor specificity protein 3 (SP3) was activated in HUVECs, responsible for Neu5Ac metabolism disorder. While RNA interference of SP3 could decrease NEU3 mRNA level and reduce endothelial inflammatory injury, indicating that SP3 might be the upstream regulatory factor of NEU3. Together, these findings identified the key role of NEU3 in regulating Neu5Ac metabolism associated with endothelial function, as well as its potential regulation mechanism. Targeting SP3/NEU3 seems to be a potential therapeutic strategy for such diseases with Neu5Ac accumulation like atherosclerosis (AS).

## 1 Introduction

Sialic acid, a nine‐carbon monosaccharide with a negative charge, has recently garnered attention as a significant marker of cardiovascular events, with N‐acetylneuraminic acid (Neu5Ac) being the predominant form in vivo [1]. Previous studies have demonstrated its critical role in promoting the progression of coronary artery diseases by activating the Rho/ROCK‐JNK/ERK pathway in vitro and in vivo [2]. Our previous work further confirmed that Neu5Ac accumulation in macrophages and endothelial cells could be an accelerator to the atherosclerosis (AS) process via inducing ferroptosis or autophagy pathways [3, 4]. These findings indicated that Neu5Ac acts as a key regulator and potential therapeutic target in the pathogenesis of cardiovascular disorders. Notably, clinical evidence has demonstrated that Neu5Ac levels are significantly elevated in patients with cancer [5], diabetes mellitus [6] and AS [7], supporting a close link between abnormal Neu5Ac accumulation and the development of multiple cardiovascular and metabolic disorders. Given the broad pathogenic implications of Neu5Ac dysregulation, elucidating the underlying mechanisms responsible for elevated Neu5Ac levels in these diseases is of great importance.

The levels of Neu5Ac are primarily regulated through its metabolic pathway. The precursor UDP‐N‐acetylglucosamine (UDP‐GlcNAc) is metabolised into Neu5Ac by a series of rate‐limiting enzymes, including UDP‐N‐Acetylglucosamine 2‐Epimerase/N‐Acetylmannosamine Kinase (GNE), N‐Acetylneuraminic Acid Synthase (NANS), N‐Acylneuraminate‐9‐Phosphatase (NANP). Then Cytidine Monophosphate N‐Acetylneuraminic Acid Synthetase (CMAS) catalyses Neu5Ac into active CMP‐Neu5Ac within the nucleus. Subsequently, CMP‐Neu5Ac is transported into the Golgi for further processing by the solute carrier family 35 member A1 (SLC35A1) transporter. In addition, sialidase of NEU family could directly cleave Neu5Ac from the termini of glycolipid chains [8, 9, 10]. Collectively, these steps coordinately determine the synthesis, activation, transport and metabolic homeostasis of Neu5Ac. Notably, the sialidase NEU family plays a critical role in cardiovascular pathophysiology. To date, four isoforms have been identified: lysosomal sialidase NEU1, cytoplasmic sialidase NEU2, plasma membrane‐associated sialidase NEU3 and lysosomal or mitochondrial membrane‐associated sialidase NEU4 [11, 12, 13]. Emerging evidence has demonstrated that NEU1 contributed to cardiovascular disease by modulating Neu5Ac levels on high density lipoprotein (HDL) and regulating leukocyte migration [14, 15, 16]. Meanwhile, NEU3 has been implicated in diverse pathological processes, including hepatic steatosis, pulmonary, cardiac fibrosis and AS, through the desialylation of key signalling molecules [17]. Despite these advances, the specific NEU isoform primarily responsible for regulating Neu5Ac accumulation during atherosclerotic progression remains poorly defined and requires further investigation.

Atherosclerosis is a chronic inflammatory and metabolically driven progressive disease [18, 19]. Emerging evidence has demonstrated that multiple pathogenic stimuli, including inflammatory factor TNF‐α, oxidised low‐density lipoprotein (ox‐LDL) and disturbed shear stress, could induce endothelial dysfunction. And this in turn promotes the recruitment of circulating monocytes and initiates early AS progression [20, 21]. Given the central role of endothelial injury in the onset and development of AS, exploring the underlying mechanisms governing endothelial dysfunction is critically important for preventing the initiation and progression of atherosclerotic lesions. Our previous study demonstrated that TNF‐α triggers disrupted Neu5Ac homeostasis in endothelial cells, implying a close link between endothelial inflammatory injury and dysregulated sialic acid metabolism [3]. Additionally, recent evidence has indicated that TNF‐α is positively correlated with the expression of sialidases like NEU3 in human dendritic cells and T cell lines [22, 23]. These findings support the hypothesis that sialidase NEU3 may participate in endothelial inflammatory dysfunction by modulating Neu5Ac metabolism, thereby potentially affecting endothelial function and subsequent atherosclerotic progression.

Here, we identified NEU3 as a key enzyme that regulates free Neu5Ac levels in inflammatory endothelial cells and atherosclerotic lesions of high‐fat diet‐fed ApoE^−/−^ mice. We demonstrated that TNF‐α promotes the release of free Neu5Ac partly through upregulating NEU3 expression, thereby contributing to endothelial dysfunction. Knockdown of NEU3 by siRNA in HUVECs reversed the aberrant elevation of Neu5Ac and reduced the expression of endothelial injury markers, including ICAM‐1 and VCAM‐1. Mechanistically, the activation of transcription factor specificity protein 3 (SP3) was partially responsible for the increased expression of NEU3, which in turn led to disturbed Neu5Ac homeostasis and impaired endothelial function.

In conclusion, NEU3 contributes to endothelial inflammatory injury via SP3‐dependent mechanism, thereby participating in the pathogenesis of AS by modulating Neu5Ac homeostasis. Strategies for targeting NEU3 or reducing Neu5Ac accumulation may represent a promising way to prevent the initiation and progression of atherosclerosis.

## 2 Materials and Methods

### 2.1 Materials and Reagents

TNF‐α (300‐01A, Peprotech), Cell Count Kit 8 (CCK‐8) (C0037, Beyotime, China), DAPI (C1005, Beyotime, China), PBS (AR0031, Boster), RIPA buffer (P0013B, Beyotime), PMSF (ST506, Beyotime), 5× loading buffer (Beyotime), Enhanced chemiluminescence western blotting system (ECL) (BL520A, Biosharp China), Oseltamivir (HY‐17016, MedChemExpress), Zanamivir (Z124915, Aladdin), SPD304 (HY‐111255A, MedChemExpress).

### 2.2 Cell Culture

Human umbilical vein endothelial cells (HUVECs) were obtained from ScienCell Research Laboratory Inc. (USA) and cultured in DMEM medium supplemented with 10% fetal bovine serum, 1% antibiotics (penicillin/streptomycin/amphotericin) in 5% CO_2_ at 37°C. THP‐1, human monocyte cell line, purchased from BeNa Culture Collection (China), was cultured in RPMI 1640 medium supplemented with 10% fetal bovine serum, 1% antibiotics (penicillin/streptomycin/amphotericin) at 37°C in a 5% CO_2_ incubator.

### 2.3 Animal Model

Eight‐week‐old male ApoE^−/−^ mice (C57BL/6J background, 20–25 g) were purchased from Beijing Vital River Laboratory Animal Science and Technology Co. Ltd. (Licence No.: SCXK (Jing) 2021‐1160006). The mice were placed in a sterile room with temperature (20°C–26°C) and humidity (50%–60%). In this experiment, mice had free access to food and water. After 1 week of acclimatisation feeding, the ApoE^−/−^ mice were randomly divided into 2 groups, a chow‐fat diet group (*n* = 8) and a high‐fat diet group (*n* = 8) fed for 8 weeks. At the end of the study, mice were sacrificed and cardiac puncture was performed by perfusing the vessels with sterile phosphate‐buffered saline (PBS)–sodium heparin solution to flush the heart and blood from the vessels. The heart was excised and fixed with 4% paraformaldehyde. Aorta, liver and kidney were collected in sterile PBS solution for further use.

### 2.4 Analysis of Aortic Lesions

Mouse aortas were taken, fixed in 4% paraformaldehyde solution and stained with Oil‐red O (ORO) (Solarbio, China) to quantify the lesion area. Paraffin‐embedded aortas were cut into 8 μm‐thick sections, stained with haematoxylin–eosin (HE) and Masson staining to visualise the necrotic core area and collagen content. Plaque images were captured under a Leica microscope and quantitatively analysed using Image‐Pro Plus (Media Cybernetics, USA). For immunostaining, aortic sections were fixed in ice‐cold methanol for 10 min, treated with 0.1% triton for 15 min and then closed with fetal bovine serum albumin at a constant temperature of 37°C for 1 h and then incubated with primary antibody at 4°C overnight. Sections were rinsed with PBS, incubated with secondary antibody for 1 h at room temperature and then sealed with an antifluorescence quencher containing DAPI. Images were taken under a Leica confocal laser scanning microscope.

### 2.5 Detection of Free Neu5Ac Levels In Vivo and In Vitro

Free Neu5Ac levels in cell culture media were determined using liquid chromatography‐mass spectrometry (LC–MS). The measured standard curve is shown in the Figure S1.

### 2.6 Cell Viability Assay

1 × 10^5^ cells were inoculated with a 96‐well plate and incubated at 37°C overnight. Cells were then treated with TNF‐α for 12 and 24 h, followed by the addition of Cell Count Kit 8 (CCK‐8) reagent to the cell culture medium. After incubation for 2 h, absorbance was measured at 450 nm to calculate cell viability.

### 2.7 Immunoblotting

After intervention of the cells, the cell culture medium was collected for subsequent LC–MS assay. The well plates were washed three times using PBS; RIPA buffer containing 1% PMSF was added and lysed on ice for 15 min. The lysed cells were de‐crosslinked using ultrasonic crusher at 30% power 1 s at a time for 5 s and centrifuged at 4°C. The cell lysate after sonication was centrifuged at 12,000 rpm for 10 min, the supernatant was taken and 4 times the volume of the supernatant was added to a 5× loading buffer and mixed in a metal bath at 99°C for 10 min to obtain the protein samples. Proteins were electrophoresed by SDS‐PAGE and transferred to 0.22 μm PVDF (Millipore, USA). After antigen blocking with 5% BSA, the blot was incubated with primary antibody overnight at 4°C, followed by incubation with secondary antibody or HRP‐labelled secondary antibody for 1 h at room temperature and finally the immunoreactive bands were detected using enhanced chemiluminescence western blotting system (ECL) with chemiluminescence imaging system (UVP). The grey value of protein bands was determined and quantified using image J 64 software.

### 2.8 Real Time Quantitative PCR Analysis

Total RNA was isolated from HUVEC using the Trizol method (Accurate Biology, China). Reverse transcription was performed using Evo M‐ML V Mix Kit with gDNA Clean for qPCR (A4A2739, Accurate biology). Real‐time PCR was performed using SYBR Green Realtime Master qPCR Mix (A4A2729, Accurate biology) and the relative expression values of mRNAs were calculated using the 2^−∆∆Ct^ method with GAPDH as endogenous control. The primer sequences used are shown in Table S1.

### 2.9 SiRNA and Transfection

HUVECs were inoculated in 12‐well plates at a cell density of 50%–60% for short RNA interference. Small interfering RNAs targeting NEU3 and SP3 (siNEU3 and siSP3) and scrambled siRNA (NC) were transfected into HUVECs at a concentration of 50 nM per well (Beijing Tsingke Biotech Co. Ltd.), and the sequences of the short interfering RNAs were shown in Table S2. After transfection for 6 h at 37°C using the commercial transfection reagent Lipofectamine 2000 (Thermo Fisher Scientific), the cell culture medium was changed to fresh complete medium. RNA was extracted after the 48 h of addition of transfection reagent, and protein was extracted at the 72 h.

### 2.10 Immunofluorescence Staining

The treated cells were taken out of the incubator, fixed with ice methanol at room temperature for 15 min and then closed with 5% BSA containing 0.1% Triton X‐100 (T8022, Solarbio) for 1 h at 37°C. Then, the cells were immunolabelled with primary antibody (ratio of 1:100) overnight at 4°C. After three washes with PBS, the cells incubated with the corresponding Alexa Fluor 555 coupled secondary antibody (1: 200) was incubated for 1 h at 37°C. Staining was performed with DAPI for 5 min at room temperature. In tissue immunofluorescence. After feeding a high‐fat diet, aortic arches and sinuses of ApoE^−/−^ mice were collected, fixed with 4% paraformaldehyde (BL539A, Biosharp) and permeabilised with 0.1% Triton X‐100. The membranes were subsequently closed at 37°C in 5% fetal bovine serum for 1 h. The membranes were stained with CD31 (ab119341 abcam China) and NEU3 (27879‐1‐AP, 1:100) primary antibodies. The remaining steps were the same as for cytologic immunofluorescence staining. Fluorescence signals were observed with a laser confocal scanning microscope and fluorescence intensity was quantified using image J.

### 2.11 Monocyte Adhesion Assay

Cells were cultured after TNF‐α treatment, and THP‐1 cells were labelled with Calcein AM (C2012‐0.1 mL, Beyotime, China) and incubated for 1 h on monolayer HUVECs. Suspended cells were washed three times with phosphate‐buffered saline. Monocyte adhesion was observed using an Olympus inverted fluorescence microscope.

### 2.12 Analysis of Differentially Expressed Genes (DEGs)

Gene expression data in plaques of patients with atherosclerosis were obtained in the Gene Expression Omnibus (GEO) database. Relevant gene expression data were analysed and extracted online using GEO2R (https://www.ncbi.nlm.nih.gov/), and difference and correlation analyses were performed in GraphPad Prism 8.0.2.

### 2.13 Luciferase Reporter Assay

The sequences of NEU3 3′UTR containing the wild‐type (WT) or mutant (Mut) bindi ng site of hsa‐miR‐637 were devised and synthesised by GenePharma (Shanghai, China). 293T were co‐transfected with the corresponding plasmids and hsa‐miR‐637 mimics/miR‐N C or hsa‐miR‐637 inhibitors/inh‐NC with Lipofectamine 2000 (Invitrogen, Carlsbad, CA, USA). To construct of a luciferase reporter gene vector containing NEU3 promoter, the full‐length NEU3 promoter containing wide or mutant type was respectively cloned into pGL3‐basic vectors (Ge necreate, Wuhan, China) and co‐transfected with or without SP3 overexpression vector later. After 48 h of incubation, the activities of firefly and Renilla luciferase were measured using the Dua l Luciferase Reporter Assay Kit (Promega, Madison, WI, USA).

### 2.14 Statistical Analysis

Statistical comparisons between the two independent groups were made using Student's *t*‐test, and one‐way ANOVA (one‐way ANOVA) was used to evaluate the statistical differences between the two groups. Data were analysed using GraphPad Prism 8.0.2 software and are presented as means ± SD. *p < 0.05; **p < 0.01, ***p < 0.001.

## 3 Result

### 3.1 Elevated Neu5Ac Were Highly Associated With Endothelial Inflammation

**FIGURE 1:**
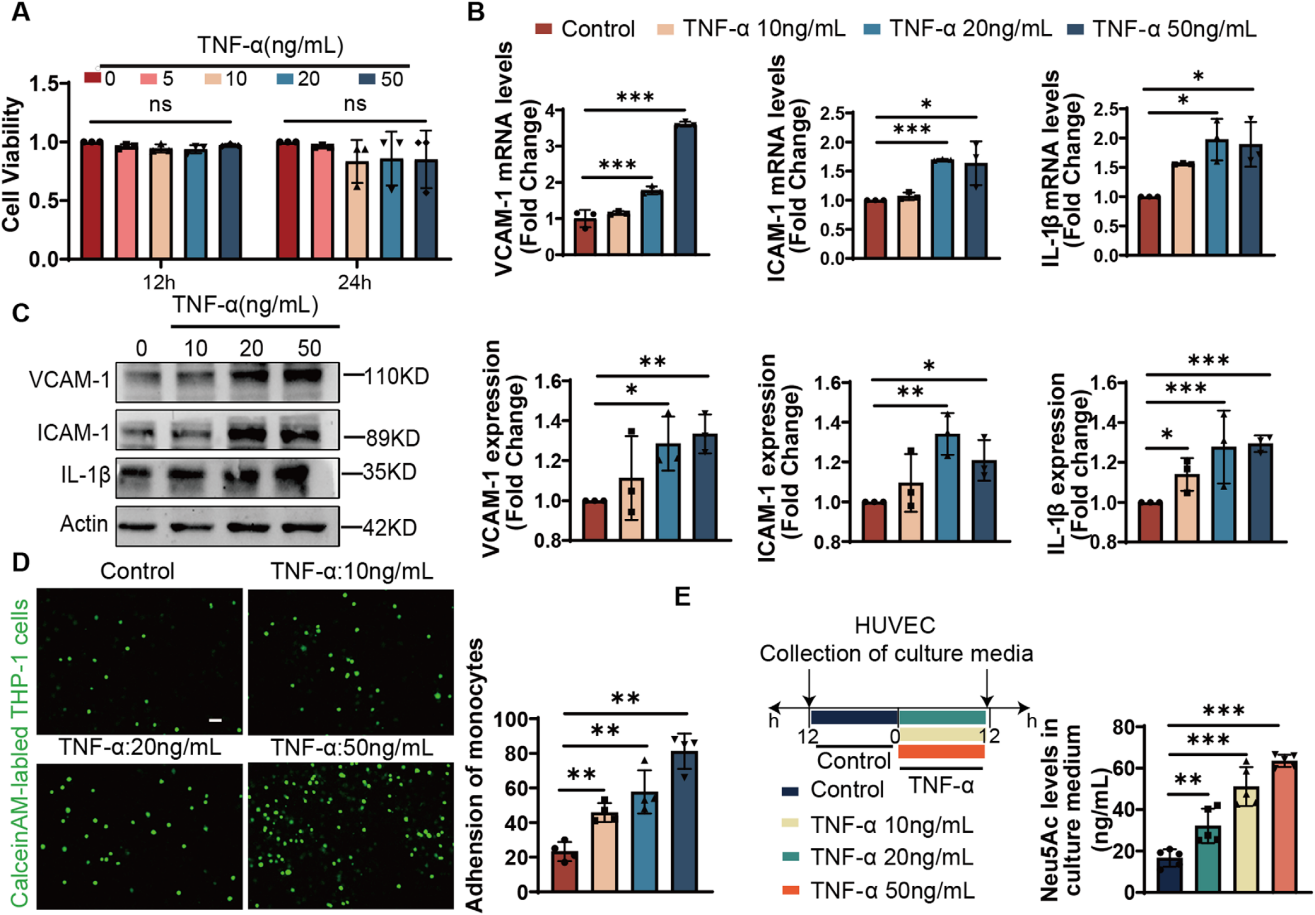
Elevated Neu5Ac were highly associated with endothelial inflammation. (A) TNF‐α (0, 5, 10, 20, 50 ng/mL) stimulated HUVEC for 12 h (left) and 24 h (right) and the effect on cell viability was detected by CCK‐8 (*n* = 3). (B–D) The mRNA and protein expression levels of VCAM‐1, ICAM‐1 and IL‐1β, and representative images of monocyte adhesion after 12 h of TNF‐α (0, 10, 20, 50 ng/mL) treatment of HUVEC (*n* = 3). (E) LC–MS analysis Neu5Ac levels in the culture medium after treatment of HUVEC with TNF‐α (0, 10, 20, 50 ng/mL) were (*n* = 5). Comparison of experimental data was performed by one‐way ANOVA; Turkey test was used. n.s. indicates that the difference is not statistically significant; ***p < 0.001, **p < 0.01, *p < 0.05.

To investigate the alterations of free Neu5Ac levels during endothelial dysfunction, we established a model of endothelial inflammatory injury induced by TNF‐α. Initially, we evaluated the impact of TNF‐α on cell viability by treating HUVECs with concentrations ranging from 5 to 50 ng/mL. Compared to the control group, none of the differences were obtained on cell viability from the TNF‐α treating group (Figure 1A). Next, we evaluated the expression of endothelial inflammatory markers. Results from Figure 1B,C showed that the mRNA and protein expression levels of VCAM‐1, ICAM‐1 and Interleukin‐1β (IL‐1β) were both increased after TNF‐α stimulation compared to the control group. The number of monocytes adhering to endothelial cells also exhibited a dose‐dependent increase when compared to the control group (Figure 1D). Furthermore, we detected the concentration of free Neu5Ac in culture medium utilising the LC–MS method. The results showed that TNF‐α induced the increase of free Neu5Ac in a concentration‐dependent manner compared to the control group (Figure 1E). Together, these findings indicated that elevated Neu5Ac levels were highly associated with endothelial inflammatory injury.

### 3.2 Upregulation of Sialidase NEU3 Involved in Endothelial Injury and AS Progression

**FIGURE 2:**
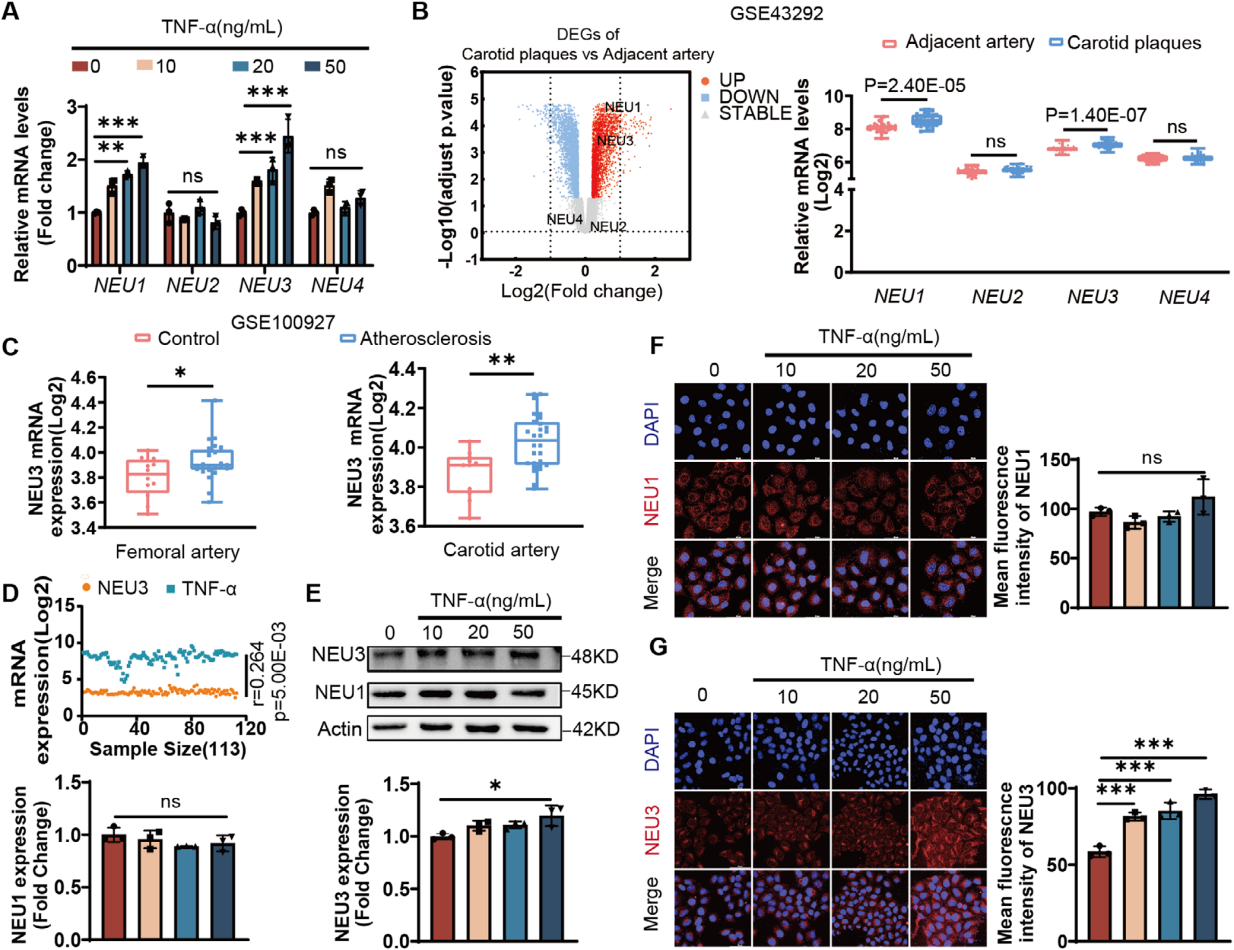
Upregulation of sialidase NEU3 involved in endothelial injury and AS progression. (A) The mRNA levels of Neu5Ac metabolism‐related enzymes after TNF‐α (0, 10, 20, 50 ng/mL) treatment of HUVEC for 12 h (*n* = 3). (B) Differential expression of Neu5Ac metabolising enzyme genes between normal adjacent tissue and atherosclerotic plaque via GEO analysis. (C) The mRNA levels in different artery tissues based on the GEO database (GSE100927). (D) The correlation analysis of NEU3 and TNF‐α in atherosclerotic plaques based on the GEO database (GSE24495) (*n* = 113). (E) The protein expression levels of NEU1 and NEU3 (*n* = 3). (F, G) Representative images of immunofluorescence staining of NEU1, NEU3 (*n* = 3) Scale bar: 50 μm. Comparison of experimental data was performed by one‐way ANOVA; Turkey test was used. n.s. indicates that the difference is not statistically significant; ***p < 0.001, **p < 0.01, *p < 0.05. UP, DOWN, STABLE represent the expression of up‐regulated genes, down‐regulated genes and non‐differentiated genes, respectively.

To investigate the mechanism underlying the elevation of free Neu5Ac levels during the endothelial inflammation process, we first assessed the expression of sialidase NEUs in HUVECs following TNF‐α treatment. As shown in Figure 2A, TNF‐α treatment significantly upregulated the expression of NEU1 and NEU3 in HUVECs compared to the control group, whereas the expression of NEU2 and NEU4 displayed no changes. To validate these in vitro findings in clinical samples, we analysed public microarray data from human carotid plaques (GSE43292). Consistent with our in vitro results, this dataset confirmed that both NEU1 and NEU3 were significantly increased in carotid plaques (Figure 2B). Further analysis of GEO data from peripheral arterial atherosclerosis (GSE100927) showed that the NEU3 mRNA levels were increased significantly in atherosclerotic plaques compared to neighbouring normal tissues (Figure 2C). Furthermore, correlation analysis using the GEO database (GSE24495) demonstrated a strong positive correlation between NEU3 expression and TNF‐α levels in atherosclerotic plaques (Figure 2D). Next, the protein expression of NEU3 was found to be upregulated after TNF‐α treatment, consistent with its mRNA level. In contrast, NEU1 protein levels remained unchanged after TNF‐α treatment(Figure 2E,F). Additionally, immunofluorescence staining further confirmed the increased expression of NEU3 in endothelial cells upon TNF‐α stimulation(Figure 2G). Collectively, these findings suggested that NEU3 upregulation is closely involved in TNF‐α induced endothelial inflammatory injury, whereas NEU1 may not play a major role in this process.

### 3.3 NEU3 Exacerbates Endothelial Inflammation Through Affecting Neu5Ac Levels

**FIGURE 3:**
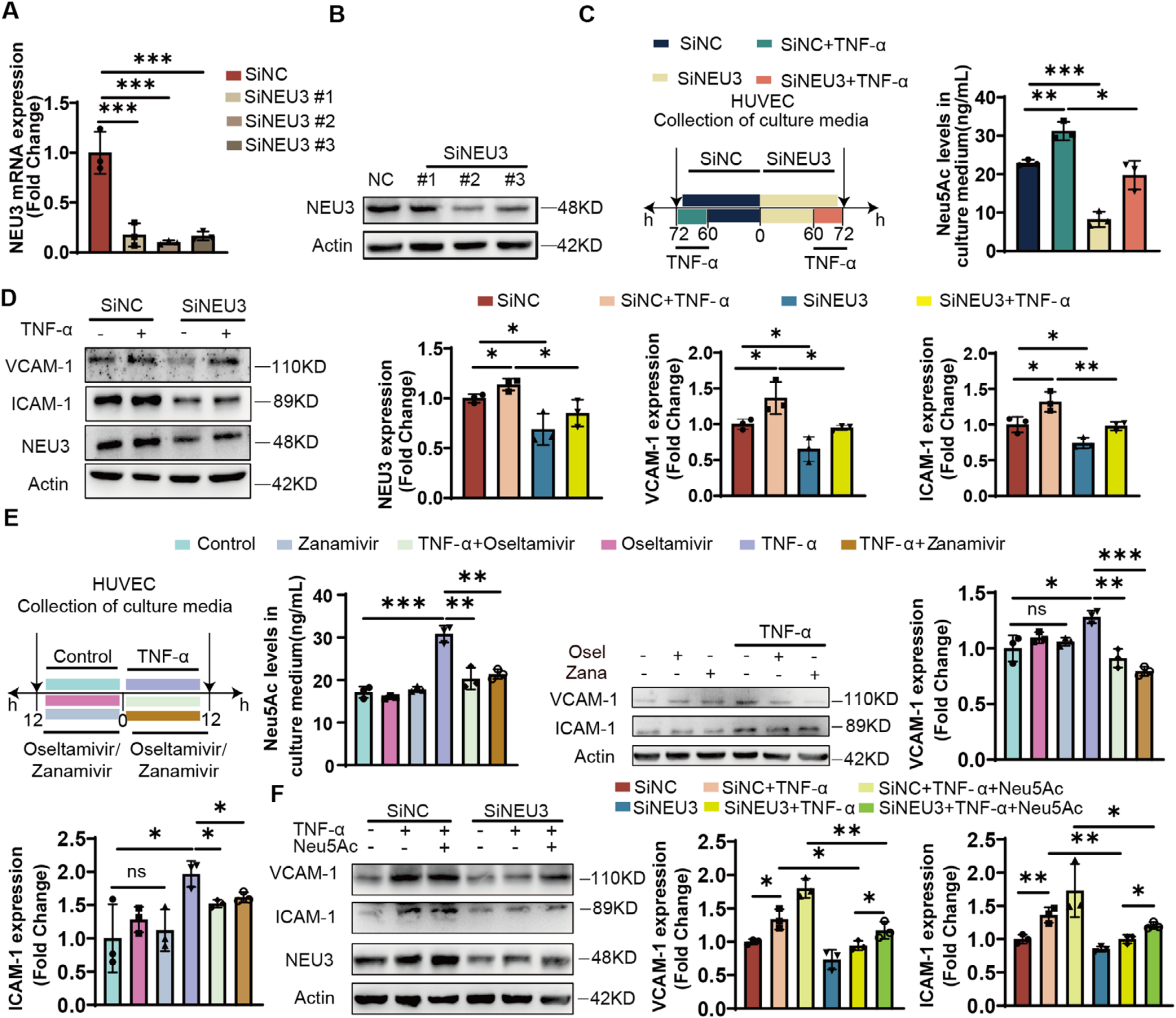
NEU3 exacerbates endothelial inflammation through affecting Neu5Ac levels. (A, B) The mRNA (*n* = 3) and protein levels (*n* = 3) were assayed in HUVECs with transfection of NEU3 siRNA for 48 and 72 h respectively. (C) LC–MS analysis of Neu5Ac level in culture medium of cells with transfection of NEU3 siRNA (*n* = 3). (D) Protein levels of VCAM‐1, ICAM‐1 and NEU3 after transfection with NEU3 siRNA and treatment with TNF‐α (50 ng/mL) (*n* = 3). (E) Neu5Ac level in the culture medium detected by LC–MS after treated with Oseltamivir (2 μM)/Zanamivir (0.5 mg/mL) and protein levels of VCAM‐1 and ICAM‐1 after treated with Oseltamivir/Zanamivir. (F) Protein levels of VCAM‐1 and ICAM‐1 after treated with supplement with exogenous Neu5Ac (2 mM) to HUVECs which transfected with NEU3 siRNA and TNF‐α (50 ng/mL) (*n* = 3). Experimental data were compared by one‐way ANOVA; Turkey test was used. n.s. indicates that the differences were not statistically significant; ***p < 0.001, **p < 0.01, *p < 0.05. NC indicates siRNA‐treated negative control group. Osel, Oseltamivir; Zana, Zanamivir.

To further verify the regulation of NEU3 in endothelial inflammation, we firstly applied three siRNA NEU3 knockdown sequences to HUVECs. Results from RT‐PCR as well as Western blot analyses confirmed NEU3 expression was successfully silenced in HUVECs (Figure 3A,B). Then we treated cells with TNF‐α after NEU3 silence. As shown in Figure 3C,D, compared with the TNF‐α group, NEU3 silencing significantly reduced Neu5Ac level and markedly decreased the protein levels of VCAM‐1 and ICAM‐1 in HUVECs. Next, we utilised NEU inhibitors oseltamivir and zanamivir to further validate the above results. As shown in Figure 3E, NEUs inhibitors could reduce Neu5Ac levels under endothelial inflammation and further decrease the expression of VCAM‐1 and ICAM‐1. These results confirmed that NEU‐mediated Neu5Ac dysregulation contributes to endothelial injury. Furthermore, we applied Neu5Ac combined with TNF‐α to detect its effect on endothelial inflammation. Compared to the control group, Neu5Ac could induce endothelial inflammatory injury and potentiated the pro‐inflammatory effect of TNF‐α. Notably, silencing NEU3 significantly decreased the protein levels of VCAM‐1 and ICAM‐1 in cells with TNF‐α treatment. However, these markers were elevated when Neu5Ac was added to NEU3‐silenced cells (Figure S2; Figure 3F). Together, these results offered evidence that NEU3 participates in TNF‐α‐induced endothelial injury through a mechanism that directly modulates free Neu5Ac levels.

### 3.4 Endothelial NEU3 Involved in Atherosclerosis Progression

**FIGURE 4:**
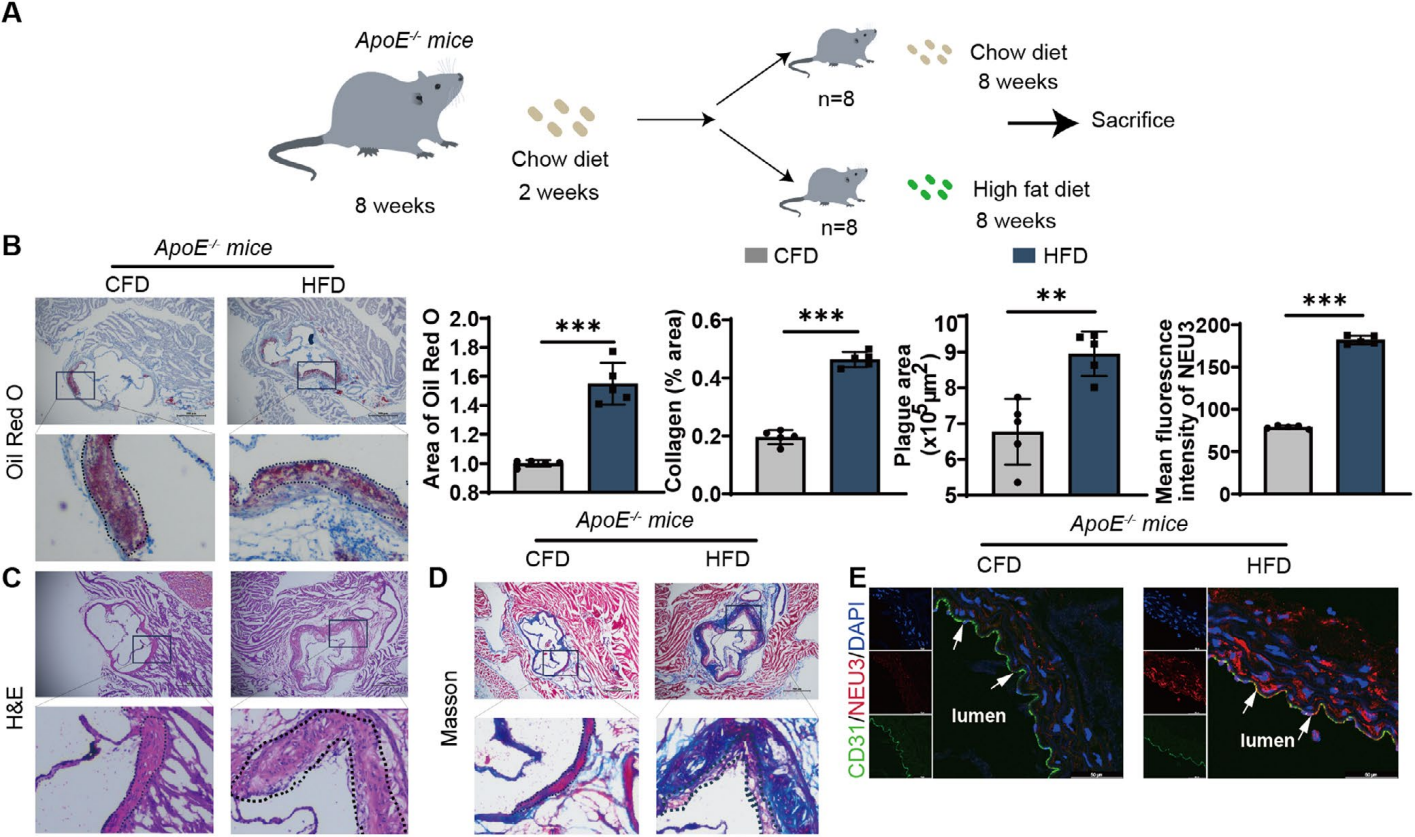
Endothelial NEU3 involved in atherosclerosis progression. (A) Schematic diagram of the atherosclerosis mouse model construction program. (B–D) Representative microscopic images of aortic sinus Oil red O, H&E and Masson staining. Scale bar: 500 μm (*n* = 5). (E) Representative microscopic images of aortic arch endothelial NEU3. Scale bar: 50 μm (*n* = 5). Experimental data were compared by one‐way ANOVA; Turkey test was used. ***p < 0.001, **p < 0.01.

To further investigate the role of NEU3 in AS process, we generated an atherosclerosis model in ApoE^−/−^ mice by feeding with high‐fat diet (HFD) for 8 weeks (Figure 4A). As shown in Figure 4B–D, the mice in HFD group exhibited higher lipid deposition, increased plaque necrotic area and increased collagen content compared to ApoE^−/−^ mice fed with chow‐fat diet (CFD), indicating that AS model has been established successfully. Next, we detected the expression of endothelial NEU3 in aortic arch by using immunofluorescence staining. As shown in Figure 4E, the level of endothelial NEU3 in HFD group was significantly up‐regulated compared to that in CFD group. Collectively, these in vivo findings are consistent with our previous in vitro results, which showed that NEU3 is significantly upregulated in TNF‐α‐induced inflamed endothelial cells. Together, these data indicated that the significant upregulation of endothelial NEU3 in atherosclerotic plaques is one of the key factors potentially contributing to the imbalance of sialic acid metabolism during AS progression, further confirming that NEU3 plays a crucial role in linking endothelial inflammation, sialic acid dysregulation and AS pathogenesis both in vitro and in vivo.

### 3.5 Transcription Factor SP3 Regulated NEU3 Expression

**FIGURE 5:**
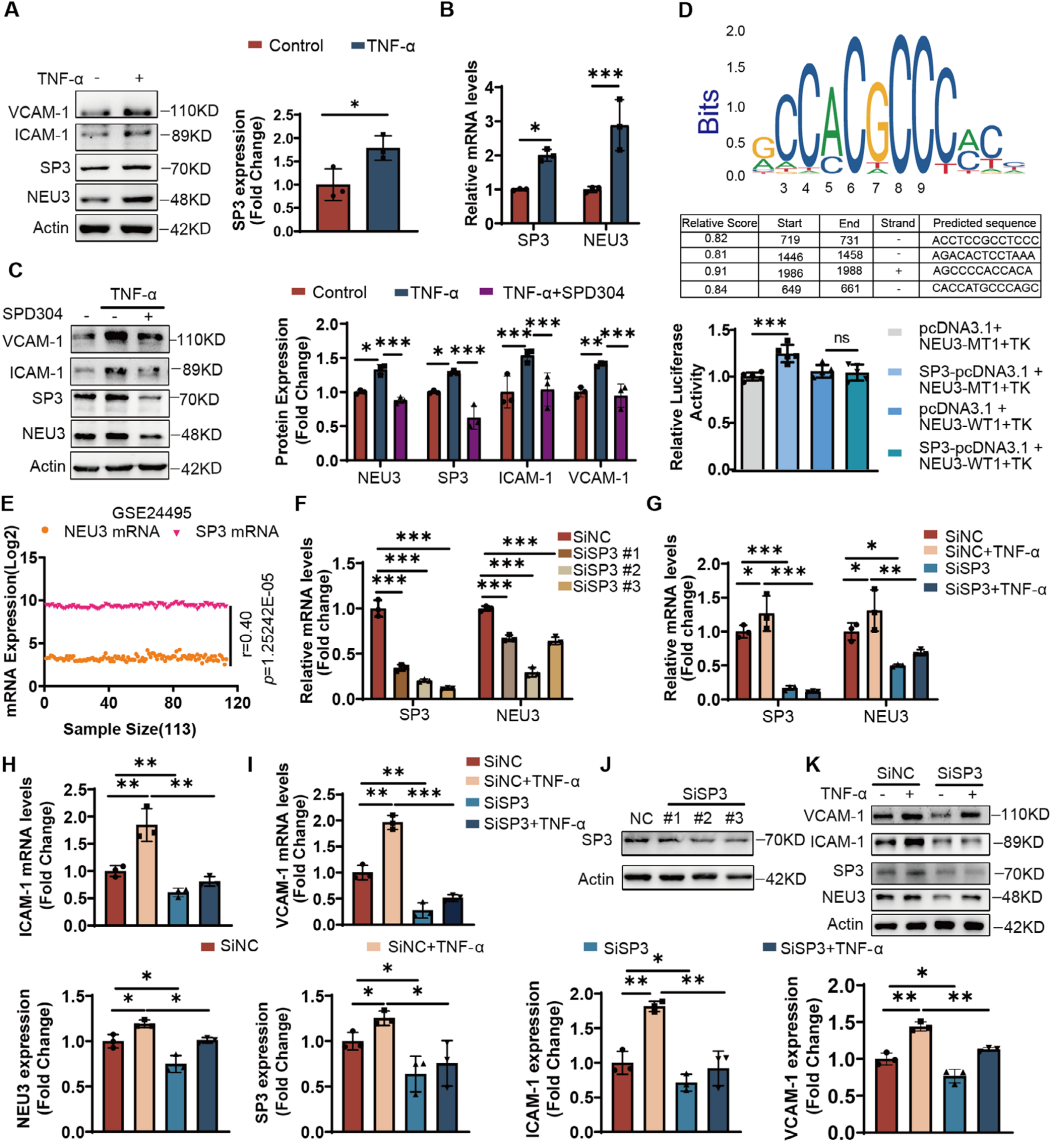
Transcription factor SP3 regulated NEU3 expression. (A, B) The mRNA and protein expression levels of SP3 under endothelial inflammation (*n* = 3). (C) The protein expression levels of SP3 and NEU3 after addition of TNFR inhibitor SPD304 (2 μM). (D) JASPAR predicted the binding motifs between NEU3 and SP3 and the relative luciferase activity after human NEU3 promoter luciferase reporter (NEU3‐MT1‐TK) or (NEU3‐WT1‐TK) was cotransfected with SP3 plasmids in 293T cells for 24 h (E) Correlation analysis of NEU3 and SP3 in atherosclerotic plaques based on the GEO database (GSE24495) (*n* = 113). (F, J) The mRNA and protein expression of SP3 and NEU3 after transfection of SP3 siRNA (*n* = 3). (G– I, K) The mRNA and protein expression levels of SP3, NEU3, VCAM‐1 and ICAM‐1 after transfection with SP3 siRNA stimulated with TNF‐α (50 ng/mL). Experimental data were compared by one‐way ANOVA; Turkey test was used. n.s. indicates that the differences were not statistically significant; ***p < 0.001, **p < 0.01, *p < 0.05. *r* indicates correlation efficient.

It has been reported that NEU3 protein level may be regulated by transcription factor SP3 in cancer [24]. However, whether SP3 exerts a similar regulatory effect on NEU3 expression during endothelial injury remains unclear. Therefore, we examined the regulatory association between SP3 and NEU3 in inflamed endothelial cells. As shown in Figure 5A,B, we found that the expression of SP3 was markedly up‐regulated together with NEU3 in HUVECs after TNF‐α treatment. The addition of TNF receptor inhibitors, SPD304, could reduce the expression of SP3 and NEU3. At the same time, SPD304 also down‐regulated the expression of endothelial inflammatory makers (Figure 5C), indicating that activation of the TNF‐α pathway is closely involved in the NEU3‐associated Neu5Ac disorder. To explore the potential molecular mechanism underlying SP3‐mediated NEU3 regulation, we predicted the potential binding sites between SP3 and the NEU3 promoter sequence, as well as their relative scores, using the Jaspar website. The results showed that the relative scores were above 0.8, suggesting that SP3 could bind to the NEU3 promoter region and drive its transcription. Furthermore, luciferase reporter assays confirmed that SP3 regulates the transcriptional level of NEU3 (Figure 5D). In addition, the published dataset from human carotid plaque (GSE24495) confirmed a strong positive correlation between NEU3 and SP3 in AS plaque (Figure 5E). Next, we constructed siRNA sequences targeting SP3 to confirm its regulation of NEU3 (Figure 5F). Compared with the TNF‐α group, SP3 knockdown significantly reduced the mRNA and protein levels of NEU3. Consistent with the down‐regulation of NEU3, the protein levels of VCAM‐1 and ICAM‐1 were also significantly decreased in SP3‐silenced cells, as shown in Figure 5G–K. These results demonstrated that SP3 knockdown alleviates TNF‐α‐induced NEU3 upregulation and subsequent endothelial inflammatory injury.

**FIGURE 6:**
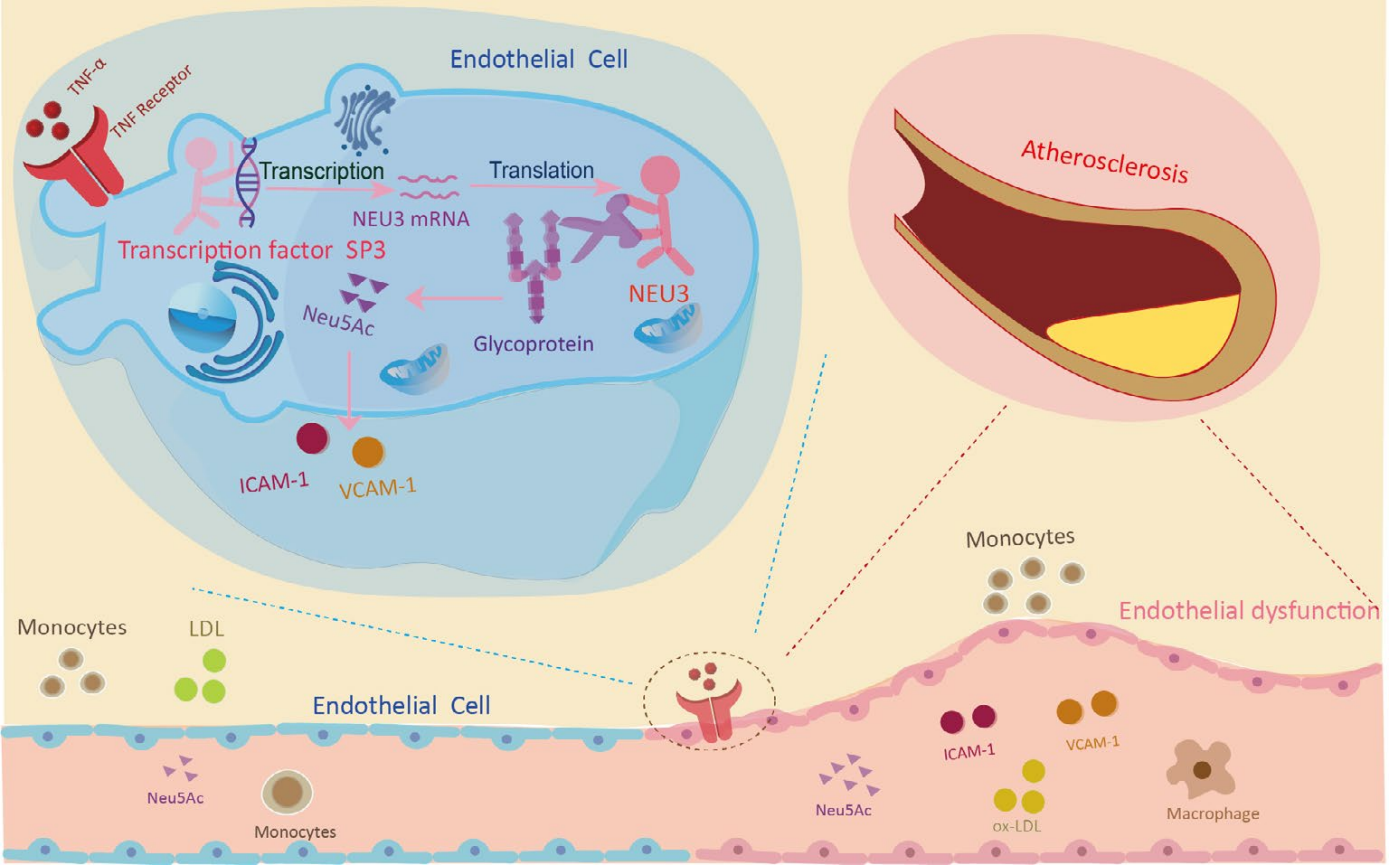
Schematic representation of induction of Neu5Ac‐associated vascular endothelial injury via NEU3 activation by transcription factor SP3. Here, we found that under TNF‐α‐induced endothelial inflammatory injury, the transcription factor SP3 is significantly activated. Upon activation, SP3 exerts a positive regulatory effect on the sialidase NEU3: It binds to the NEU3 promoter region to drive NEU3 transcription and upregulate its expression. Elevated NEU3 expression further catalyses the cleavage of terminal Neu5Ac residues from glycoproteins and glycolipids, leading to a significant increase in intracellular and extracellular free Neu5Ac levels. This dysregulation of Neu5Ac homeostasis, in turn, exacerbates the process of endothelial inflammatory injury—ultimately contributing to the initiation and progression of atherosclerosis.

Collectively, these findings clearly indicated that SP3 positively regulates NEU3 expression in inflamed endothelial cells: TNF‐α activation upregulates SP3, which in turn binds to the NEU3 promoter to drive its transcription, leading to NEU3 upregulation, Neu5Ac dysregulation and ultimately endothelial inflammatory injury (Figure 6). This regulatory axis provides a novel molecular mechanism underlying Neu5Ac accumulation responsible for endothelial dysfunction during AS progression.

## 4 Discussion

Atherosclerosis starts with endothelial cell dysfunction when exposed to risk factors including inflammatory cytokines [25, 26]. Our previous study demonstrated that increased levels of Neu5Ac play a crucial role in inducing endothelial dysfunction [3, 4]. However, few studies have clearly elucidated the potential regulatory mechanism between Neu5Ac homeostasis and endothelial inflammation. Our present findings identified NEU3 as a key enzyme in promoting Neu5Ac‐associated vascular endothelial injury, facilitated by the activation through the transcription factor SP3. This discovery opens up new avenues for innovative therapeutic approaches in the treatment of disease with Neu5Ac accumulation such as AS.

Sialidases (also known as neuraminidases, NEUs) regulate physiological processes by catalysing the removal of α‐glycosidically linked Neu5Ac residues from glycoproteins and glycolipids [17]. Members of this enzyme family differ in their subcellular localisation and enzymatic properties, as well as their chromosomal localisation and exhibit tissue‐specific expression patterns [27, 28, 29, 30, 31, 32]. Among NEUs isoforms, sialidase NEU3 plays a significant role in signalling processes and its abnormal expression is closely associated with various pathological conditions. Previous studies have demonstrated that NEU3 up‐regulation is highly correlated with the malignant phenotype of several cancers, including colon, renal, ovarian and prostate cancers. Inhibiting NEU3 expression could alleviate cancer progression [33, 34, 35, 36]. Beyond cancer, NEU3 is also involved in congenital heart disorders. It triggers cellular responses to chronic hypoxia by upregulating hypoxia inducible factor‐1 (HIF‐1α) [37], and it inhibits transforming growth factor‐β (TGF‐β) signalling pathway to reduce cardiac fibrosis and attenuate inflammatory progression [38]. Collectively, these findings indicated that NEU3 is actively involved in the progression of inflammatory disease. In the context of AS, emerging evidence has provided the link NEU3 to disease pathogenesis. For instance, researchers have found that NEU3 interacts with NEU1 and then induces deacetylation of LDL, therefore promoting macrophage lipid uptake [39], suggesting an important role of NEU3 in AS progression. Additionally, Moon reported that the increased NEU3 expression reduces matrix metalloproteinase 9 (MMP‐9) expression in vascular smooth muscle cells, suggesting that NEU3 may have the potential to prevent vascular proliferation‐associated diseases [40]. Although elevated sialidase activity and Neu5Ac levels have been associated with AS progression [41, 42, 43], no available reports clarify the potential association between NEU3, Neu5Ac disorder, endothelial inflammation and AS. Our findings propose that NEU3 participates in the atherosclerotic process by trimming the final Neu5Ac residues of glycoproteins, which induces Neu5Ac accumulation and subsequently provokes endothelial inflammation.

The SP transcription factor family includes three conserved Cys2His2 zinc fingers that form sequence‐specific DNA‐binding domains [44]. Specificity protein 1 (SP1) and SP3 are transcription factors in the SP family commonly expressed in mammalian cells, and they bind to cells and act through a GC box to regulate the expression of multiple target genes [45, 46, 47, 48]. SP1 and SP3 have similar structures and highly homologous DNA‐binding domains [49]. Studies have reported that SP1 participates in various physiopathological processes, such as lipid metabolism, vascular inflammation and platelet stabilisation, by interacting with numerous vital regulators, including vascular endothelial growth factor (VEGF), angiotensin II (Ang II) and stromal cell‐derived factor‐1 (SDF‐1) [50, 51, 52]. However, few studies reported the regulation of SP3. Recently, Nguyen found that MiR‐223 could activate SP3 in macrophages from atherosclerotic mice and then enhanced inflammatory signalling pathway [53], suggesting that SP3 may be involved in inflammation regulation during AS process. Our present study further confirmed that SP3 could regulate the expression of NEU3, and then enhanced endothelial inflammatory injury through elevated levels of Neu5Ac. Together with the previous research, we extend the research gap regarding SP3's regulatory role in AS and also establish a novel regulatory axis that links sialic acid metabolism and endothelial dysfunction in atherosclerotic progression.

However, several limitations of the present study should be acknowledged. First, although our in vitro findings strongly suggest a pro‐inflammatory role of NEU3 in endothelial cells, the lack of in vivo genetic evidence, particularly from endothelial‐specific *Neu3* knockout mice, limits the definitive conclusion regarding its cell‐autonomous function in atherosclerosis progression. The murine *Neu3* gene exhibits complex alternative splicing, yielding multiple protein transcripts, which necessitates careful design to ensure complete ablation of all functional isoforms. We are currently characterising these variants to generate a rigorously validated *Neu3*‐floxed mouse line, and future studies will cross these mice with endothelial‐specific Cre drivers to assess the impact of endothelial NEU3 deficiency on atherosclerotic lesion development in vivo. Secondly, while we observed that the NEUs inhibitors oseltamivir and zanamivir attenuated endothelial inflammatory injury in vitro, suggesting their potential therapeutic benefit in AS, the present study did not extend these findings to in vivo disease models. We are going to plan such an experiment to evaluate the effects of oseltamivir and zanamivir treatment on plaque development and endothelial function in atherosclerotic mouse models. Thirdly, our preliminary evidence indicates that transcription factor SP3 regulates NEU3 expression under inflammatory conditions. However, SP3 activity is known to be modulated by various post‐translational modifications, including sumoylation, acetylation and methylation. The specific modification responsible for SP3 activation in this context remains unexplored. Future work should dissect which post‐translational event governs SP3‐mediated NEU3 transcription, which could reveal additional regulatory layers and potential intervention points.

Finally, our study presents compelling evidence demonstrating that NEU3, under the positive regulation of SP3, upregulates Neu5Ac levels, thereby intensifying endothelial inflammation and fostering the progression of atherosclerosis (Figure 6). These findings contribute to a more comprehensive understanding of the interplay between metabolic disturbances and endothelial dysfunction during atherosclerosis progression. Furthermore, they provide support for the notion that Neu5Ac can serve as a potential marker for cardiovascular disease. Consequently, NEU3 emerges as a significant therapeutic target for atherosclerosis treatment.

## Author Contributions

**Peng Xiang:** methodology, software. **Xianmin Wang:** formal analysis, supervision. **Qingqiu Chen:** methodology, formal analysis, software. **Yining Lu:** conceptualization, methodology, software, data curation, supervision, formal analysis, validation, investigation, writing – review and editing, writing – original draft. **Tongchuan Wang:** investigation, validation, formal analysis. **Chao Yu:** funding acquisition, writing – review and editing, project administration, resources. **Hu Rong:** investigation, validation, formal analysis. **Limei Ma:** funding acquisition, writing – review and editing, project administration. **Chen Le:** methodology, software.

## Funding

This work was supported by Chongqing Technology Innovation and Application Development Special Project (cstc2020jscx‐msxmX0070), Chongqing Natural Science Foundation (CSTB2023NSCQ‐MSX0416), Chongqing Talent Project (cstc2021ycjh‐bgzxm0004) and Program for Youth Innovation in Future Medicine, Chongqing Medical University (W0069).

## Conflicts of Interest

The authors declare no conflicts of interest.

## Supporting information


**Figure S1:** jcmm71169‐sup‐0001‐Supinfo.docx.

## Data Availability

The data that support the findings of this study are available on request from the corresponding author. The data are not publicly available due to privacy or ethical restrictions.
