# Supplementary material for: Sialidase Neu3 Induced Sialic Acid Disorder and Promoted Vascular Endothelial Injury Through Transcription Factor SP3
Source: J Cell Mol Med. 2026 May 12;30(9):e71169. doi: 10.1111/jcmm.71169 (PMC13161992; doi:10.1111/jcmm.71169)
Supplement: Supplementary file 1 — Figure S1: jcmm71169‐sup‐0001‐Supinfo.docx. [file JCMM-30-e71169-s001.docx]

**Supplemental Figures**

**Fig.S1**


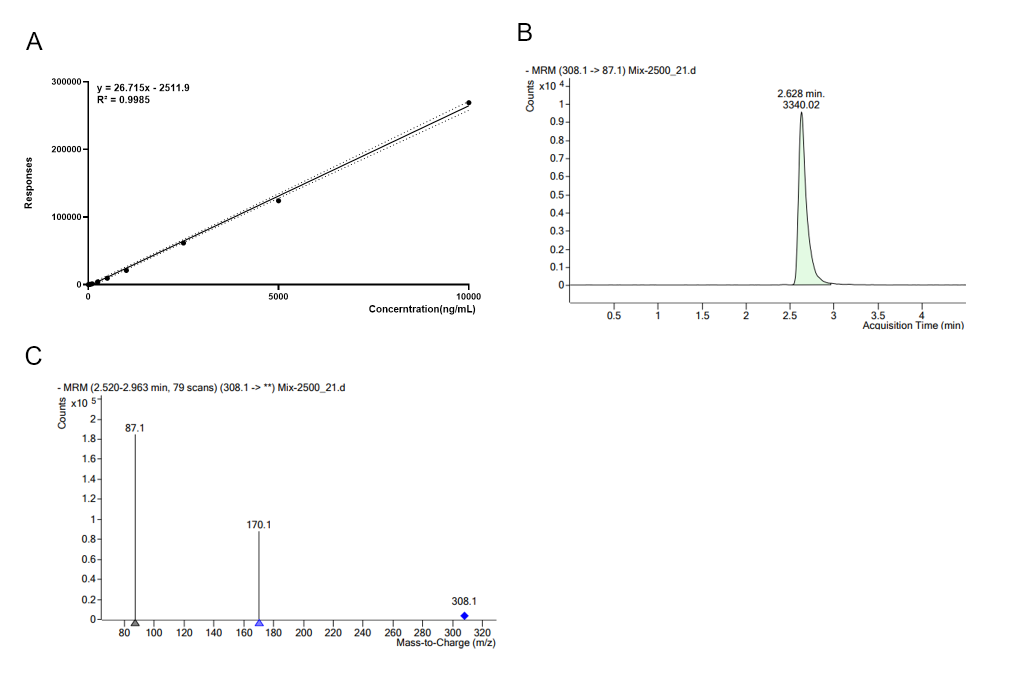


**Fig. S1 Neu5Ac levels analysis by using LC-MS**. (A) Where x is the concentration of Neu5Ac, y is the peak area of Neu5Ac, and R^2 is the regression coefficient. (B-C) Representative multiple reaction monitoring (MRM) chromatograms of N -acetylneuraminic acid (Neu5Ac) in cell culture medium.

**Fig.S2**


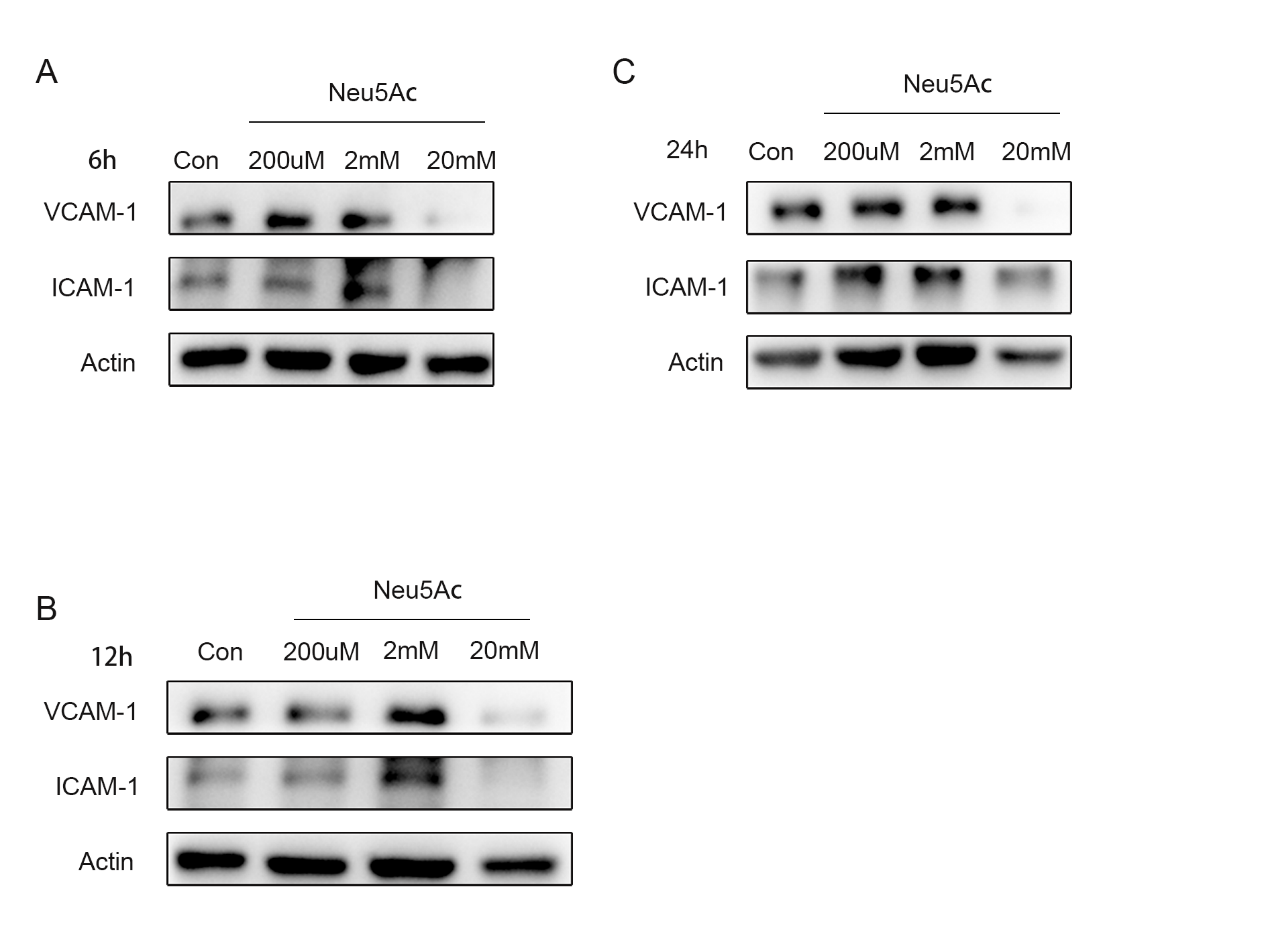


**Fig. S2 The expression levels of inflammatory cytokine proteins under different treatment conditions.** Figures A, B, and C show the changes in inflammatory cytokine protein expression levels in HUVECs treated with different concentrations of Neu5Ac for 6h, 12h, and 24h, respectively.

**Table S1**

| **Gene name** | **Primer Sequence** | **NO.** |
| --- | --- | --- |
| GAPDH | GCACCGTCAAGGCTGAGAAC | 2597 |
|  | TGGTGAAGACGCCAGTGGA |  |
| VCAM-1 | CGAACCCAAACAAAGGCAGAG | 7412 |
|  | GAGGAAGGGCTGACCAAGAC |  |
| ICAM-1 | TCTTCCTCGGCCTTCCCATA | 3383 |
|  | AGGTACCATGGCCCCAAATG |  |
| NEU3 | AAGTGACAACATGCTCCTTCAA | 10825 |
|  | TCTCCTCGTAGAACGCTTCTC |  |
| SP3 | ACAGCTACATCCAGGAGAGAATG | 6670 |
|  | ACCTCTTCCACCACCTTCTTTAC |  |
| IL-1β | TGGCAGAAAGGGAACAGAAA | 16176 |
|  | CTGGCTGATGGACAGGAGAT |  |

**Table S1** The primer sequences used in Real time quantitative PCR analysis.

**Table S2**

| **Gene name** | **Primer Sequence** |
| --- | --- |
| SiNEU3-1 | GGUUGACCUAGGUAUCUAU |
|  | AUAGAUACCUAGGUCAACC |
| SiNEU3-2 | CGCCUUUGCUUCAUCUACA |
|  | UGUAGAUGAAGCAAAGGCG |
| SiNEU3-3 | CACCAUGGUAGACUCAUUA |
|  | UAAUGAGUCUACCAUGGUG |
| SiSP3-1 | GGUGAUUCUACCUUGAAUA |
|  | UAUUCAAGGUAGAAUCACC |
| SiSP3-2 | GGUAUUCACUCUAGCAGUA |
|  | UACUGCUAGAGUGAAUACC |
| SiSP3-3 | CAAUAGUGUCGAUCUAGAU |
|  | AUCUAGAUCGACACUAUUG |

**Table S2** The sequences of the short interfering RNAs.
